# Elevated Expression of KiSS-1 in Placenta of Chinese Women with Early-Onset Preeclampsia

**DOI:** 10.1371/journal.pone.0048937

**Published:** 2012-11-08

**Authors:** Chong Qiao, Chunhui Wang, Jiao Zhao, Caixia Liu, Tao Shang

**Affiliations:** 1 Department of Obstetrics and Gynecology, Shengjing Hospital, China Medical University, Shenyang, Liaoning, China; 2 Department of Hepatobiliary Surgery, General Hospital of Shenyang Military Region, Shenyang, Liaoning, China; VU University Medical Center, The Netherlands

## Abstract

Preeclampsia (PE) is a heterogeneous syndrome affecting 2% to 8% of all pregnancies and is the world’s leading cause of fetal and maternal morbidity and mortality. In many cases of PE, shallow trophoblast invasion results in inappropriate maternal spiral artery remodeling and impaired placental function. Multiple genes have been implicated in trophoblast invasion, among which are KiSS-1 and GPR54. The gene product of KiSS-1 is metastin, which is a ligand for the receptor GPR54. Both metastin and GPR54 are expressed in the placenta of normal pregnancy and have been implicated in modulating trophoblast invasion through inhibiting migration of trophoblast cells. We have previously reported that the expression level of KiSS-1 was higher in trophoblasts from women with preeclampsia as compared to normal controls. Here, using quantitative RT-PCR, Western blot analysis and immunohistochemistry, we extend our analysis to demonstrate that elevated KiSS-1 expression occurs only in early-onset preeclampsia (ePE) and not late-onset preeclampsia (lPE). However, no difference in the expression levels of GPR54 is observed between ePE, lPE, and normal controls. Further, we show that KiSS-1 expression is also increased in placenta of intrauterine death and birth asphyxia in comparison to normal newborns of ePE and lPE. Our findings suggest that aberrant upregulation of KiSS-1 expression may contribute to the underlying mechanism of ePE as well as birth asphyxia.

## Introduction

Preeclampsia is a serious complication of pregnancy, which affects 2–8% of all pregnancies and is one of the leading causes of maternal and perinatal mortality and morbidity worldwide [1]. Although the primary mechanism of preeclampsia is still unknown, it is characterized by impaired placental function, abnormal trophoblast invasion, deficient physiologic maternal spiral artery modification, increased apoptosis of trophoblastic cells and placental ischemia. Failure of normal trophoblast invasion leads to inappropriate development of maternal spiral artery, which interferes with normal villus development and reduces placental perfusion [2], [3]. A number of factors have been implicated in trophoblast invasion, such as matrix metalloproteinases-2 and 9 (MMP-2 and MMP-9 ) [4], metastin (which is encoded by the *KiSS-1 gene),* and its receptor GPR54 [5].

**Table 1:** Clinical characteristics of pregnant women with early and late onset preeclampsia and the matched control group (Control A and B).

	Control A23–33^+6^ weeks(n = 40)	ePE23–33^+6^ weeks(n = 36)	Control B34–39 weeks(n = 40)	LPE34–39 weeks(n = 40)
**Maternal age(years)**	27.5±3.6	31.6±5.9	26.7±5.3	29.5±4.1
**Gestational age(weeks)**	29.1±2.5	28.8±3.1	37.3±1.9	37.1±2.2
**Birth weight(g)**	1078.5±438.7	1038.9±470.2	3147.2±746.1	3062.7±802.8
**Placental weight(g)**	384.2±104.7	278.8±93.7	425.2±79.3	493.4±112.4
**SBP(mmHg)**	114.5±11.6	166.9±14.7*	112.2±8.1	162.7±18.2 $
**DBP(mmHg)**	64.2±7.5	106.4±13.6*	68.0±8.2	95.1±6.8$
**Proteinuria**	N/A	++-+++	N/A	+-+++

Values are shown as mean ± SD.

PE, preeclampsia; SBP, maximal systolic blood pressure; DBP, maximal diastolic blood pressure; Control A and Control B are gestational age-matched controls of normal pregnancies for ePE and lPE, respectively.

* *P*<0.05, ePE versus Control A.

$ *P*<0.05, lPE versus Control B.

There were no significant differences in maternal age and delivery age between early and late-onset preeclampsia group and the matched control groups (*p*≥0.05). The blood pressure, systolic and diastolic blood pressures were significantly higher in preeclampsia groups than that in the matched control group (*p*<0.05).

The severity of the clinical manifestations of preeclampsia is directly related to the time of onset. An onset before 34 weeks of gestation is defined as early-onset preeclampsia (ePE), while an onset at 34 weeks or later is defined as late-onset preeclampsia (lPE) [6], [7]. It has been suggested that the early- and late-onset preeclampsia may represent distinct entities with different etiologies, rather than a single disease manifested at different stages [8], [9].

The early-onset form is more severe, frequently leading to delivery of growth-retarded premature babies or poor outcomes for the newborns and the mothers. Abnormal placental development is believed to be more strongly associated with early-onset preeclampsia, whereas late-onset preeclampsia is believed to occur secondary to maternal microvascular diseases, such as long-term hypertension, or reflect a maternal genetic disposition [10]. This distinction is further supported by the fact that placenta from late-onset subjects are morphologically similar to those from healthy pregnancies. In contrast, early-onset preeclampsia has been associated with abnormal placental morphology [11]. Despite these differences, early- and late-onset forms are not separated in most studies of preeclampsia.

The KiSS-1 gene encodes a 145 amino acid precursor peptide, which is cleaved to produce a 54 amino acid peptide, called metastin (also known as Kisspeptin or Kp-54) [12], [13]. Metastin is a ligand for the G-protein coupled receptor GPR54, both of which are expressed in the placenta of normal pregnant humans [14]. KiSS-1 is also called metastasis suppressor gene due to its ability to suppress metastasis of various tumors [15]. The metastin/GPR54 system has been shown to play an important role in repressing trophoblast invasion through control of its migratory properties [5], [16]. The interaction between metastin and GPR54 also has been implicated in controlling puberty and productivity [17].

We have previously reported that in trophoblasts from women with preeclampsia the expression levels of KiSS-1 were higher and those of MMP-9 were lower, as compared to normal controls. We also reported that there was an inverse correlation between KiSS-1 expression level and birth weight [18]. Given that the metastin/GRP54 system has been implicated in inhibiting trophoblast invasion [19] and that preeclampsia has been associated with trophoblast shallow invasion [2], we hypothesize that an abnormal metastin/GRP54 system might contribute to the pathogenesis of early-onset preeclampsia. In this report, we compare the expression levels of both KiSS-1 and GPR54 in placental tissues between preeclampsia and normal pregnancy and determine whether an upregulated expression of either or both genes correlates with early or late-onset preeclampsia.

## Materials and Methods

### Study Population

This study was conducted at the local Ethics Committee of Medical Faculty of Shengjing Hospital after institutional review board approval of tissue collection and preparation and written informed consent was obtained from each patient included in the study.

All study patients were recruited from the Shengjing Hospital, China Medical University, during the time period Jan 2009-July 2011. Only women with single pregnancies were included. Women with chronic hypertension, collagen vascular disease, any evidence of intra-partum infection or other pregnancy complications such as fetal anomalies, chromosomal abnormalities or diabetes mellitus were excluded from this study. In each case, gestational age was confirmed from the last menstrual period or, where doubt existed, by an ultrasound examination before 14 weeks of gestation.

**Figure 1:**
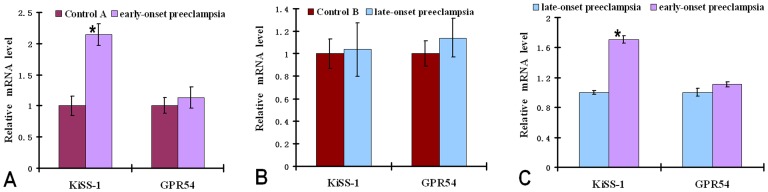
KiSS-1 and GPR54 mRNA expression in early-onset and late-onset pre-eclamptic placenta. Quantitative RT-PCR analysis of KiSS-1 and GPR54 mRNA expressions in preeclamptic (early-onset and late-onset) pregnancies compared with the respective matched control groups. The results demonstrated a significant up-regulation of KiSS-1 transcripts in the placenta of the early-onset preeclamptic pregnancies (A) and not significant difference between the late-onset preeclamptic (B) and control pregnancies. The transcript level of the KiSS-1 was significantly higher in early-onset placenta compared with late-onset preeclampsia (C). GPR54 revealed no significant difference in expression between early-onset/late-onset preeclamptic placenta and controls. Data represent means±SD after normalization to β-actin. *P<0.05, significantly decreased/increased compared with the respective control.

**Figure 2:**
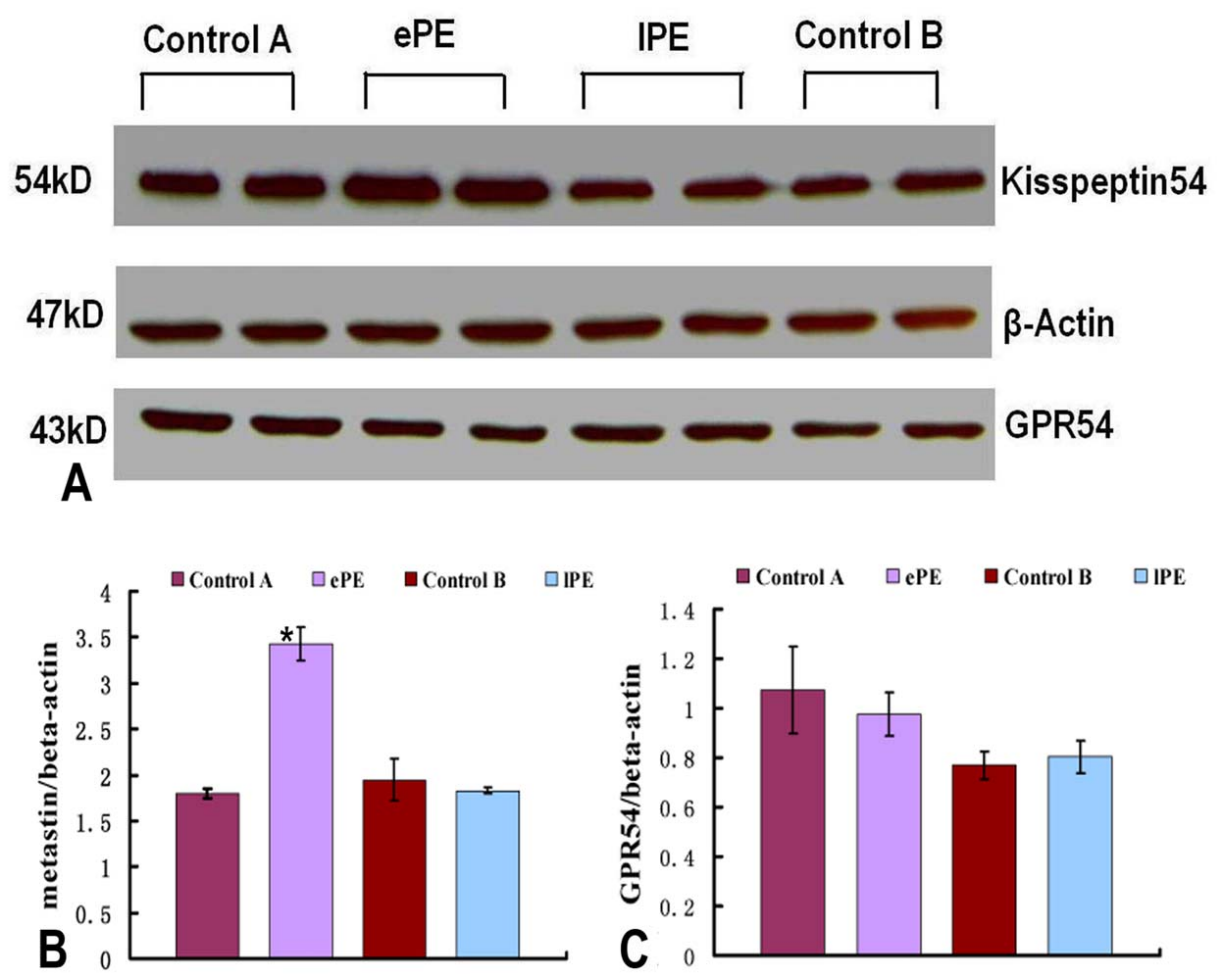
Metastin and GPR54 protein expressions in early-onset and late-onset preeclamptic placenta. (A) Representative western blot of metastin and GPR54 expressions in placenta of patients with ePE and lPE compared with the matched controls. Thirty micrograms of total protein was separated on 10% SDS-PAGE gel. β-actin was used as a loading control. (B) Densitometric analysis of metastin protein expressions of placenta samples after normalization to β-actin. Metastin protein was significantly increased in early-onset preeclamptic placenta. The late-onset preeclamptic placenta revealed no significant change in expression of metastin compared with the matched control. (C) Densitometric analysis of GPR54 expression in placenta of ePE and lPE compared with their matched controls. Neither the early-onset preeclamptic placenta nor the late-onset preeclamptic placenta revealed a significant change in expression of GPR54 compared with their respective controls. Data represent means±SD after normalization to β-actin. *P<0.05, significantly decreased/increased compared with the respective control.

Two different groups of cases were included: (1) early-onset preeclampsia (ePE), which consisted of women diagnosed and delivered before gestational week 34 (23–33^+6^ weeks, n = 36); (2) late-onset preeclampsia (lPE), which consisted of women diagnosed and delivered after gestational week 34 (34–39 weeks, n = 40). Preeclampsia was diagnosed according to international criteria [20]. Generally, preeclampsia was defined as new-onset hypertension (140/90 mmHg or greater) observed on at least 2 separate measurements 6 hours or more apart, combined with proteinuria (2 or greater on a dipstick or in a 24-hour urine sample showing 300 mg/24 hour or greater) in the second half of pregnancy in a previously normotensive woman.

Antihypertensive treatment was commenced in both groups of women with preeclampsia if the systolic blood pressure rose above 170 mmHg, if the diastolic rose above 110 mmHg, or both conditions existed.

According to the guidelines proposed by the American Academy of Pediatrics (AAP) and the American College of Obstetricians and Gynecologists (ACOG), we diagnosed newborns with birth asphyxia when all the followings were present: umbilical artery pH<7.0, APGAR score<4 at 5 minutes, multi-organ failure, and evidence of hypoxic-ischemic encephalopathy. Normal newborn, birth asphyxia, and intrauterine fetal death (IUD) were 21,11 and 4 cases in ePE, respectively. Normal newborn and birth asphyxia were 36 and 4 cases in lPE, respectively.

**Figure 3:**
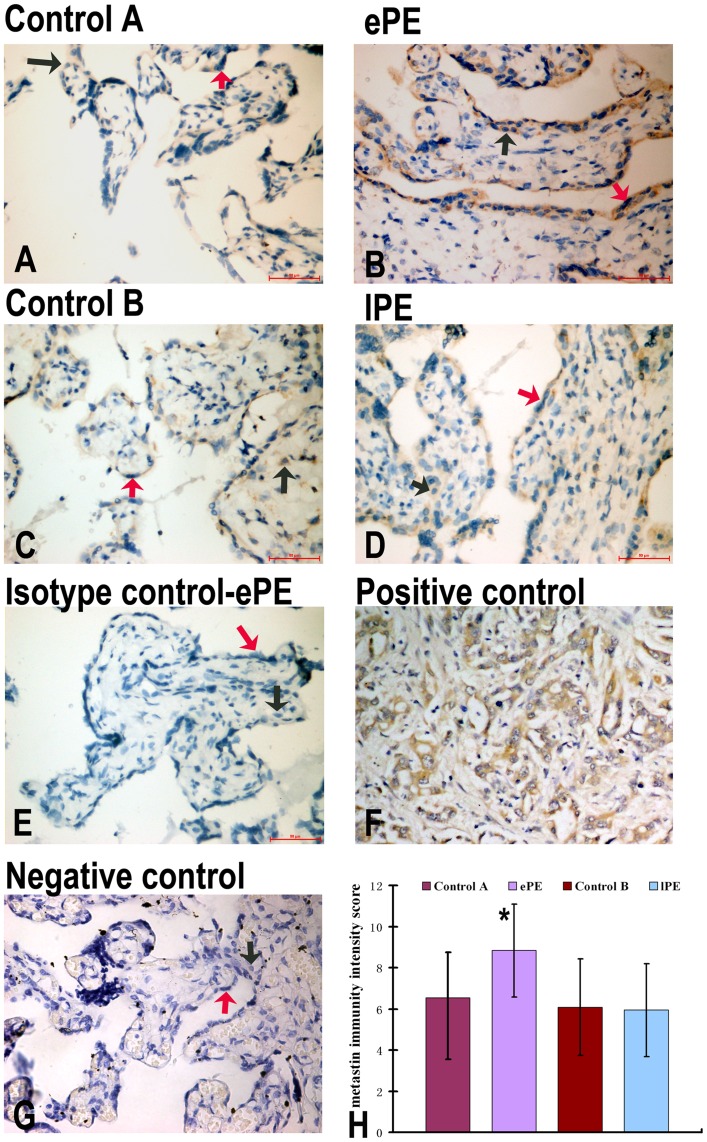
IHC of metastin expression in the placentas of control A (A), ePE (B), control B (C), lPE (D), isotype control (E), positive control (F), and negative control (G). Panel H shows metastin immunity intensity score semiquantitation. Metastin staining is present in both syncytiotrophoblasts and cytotrophoblasts from Control A, ePE, Control B and lPE (A–D). Trophoblasts from ePE had higher expression of metastin as compared to control A (A, B). lPE had the same intensity expression of metastin as compared with control B (C,D). ePE had significantly higher expression of metastin as compared with control A and lPE (H). Red arrows indicate the syncytiotrophoblast; Black arrows indicate the cytotrophoblast.

Because the gestational age was different between the ePE and lPE groups, their respective gestational age-matched groups were used as controls (control A: 23–33^+6^weeks, n = 40 and control B: 34–39 weeks, n = 40). For the control group, women with chronic hypertension, renal disease, collagen vascular disease, any evidence of intrapartum infection or other pregnancy complications such as fetal anomalies or chromosomal abnormalities were excluded from this study. Those women recruited in Control A gave birth due to cervical incompetence. Intra-uterine infection was excluded based on WBC counting and pathological examination of fetal membrane. In Control A, there were 38 live birth and 2 stillbirth, and no birth asphyxia occurred in this group. The average birth weight was 1078.5±438.7 g. Those 38 live newborns were transferred to NICU in our hospital and discharged 1 to 3 months later. The Clinical characteristics of patients are summarized in Table 1.

### Placental Tissues Collections

Immediately after delivery, the cord was clamped, and each placenta was cut. For RNA and protein isolation, only chorionic tissue from the central part of the placenta was collected and contamination with maternal decidua and amniotic membranes was excluded by morphological observation. Tissues were rinsed by saline for 3 times, which were treated by DEPC. Tissues were snap frozen in liquid nitrogen immediately and stored at –80°C until extraction of matched RNA and protein samples. For immunohistochemical staining, tissue samples were fixed immediately after removal in 10% neutral buffered [in phosphate-buffered saline (PBS), pH 7.0] formalin for 24 h at room temperature, dehydrated in a series of ethanol and xylene, and then embedded in paraffin.

**Figure 4:**
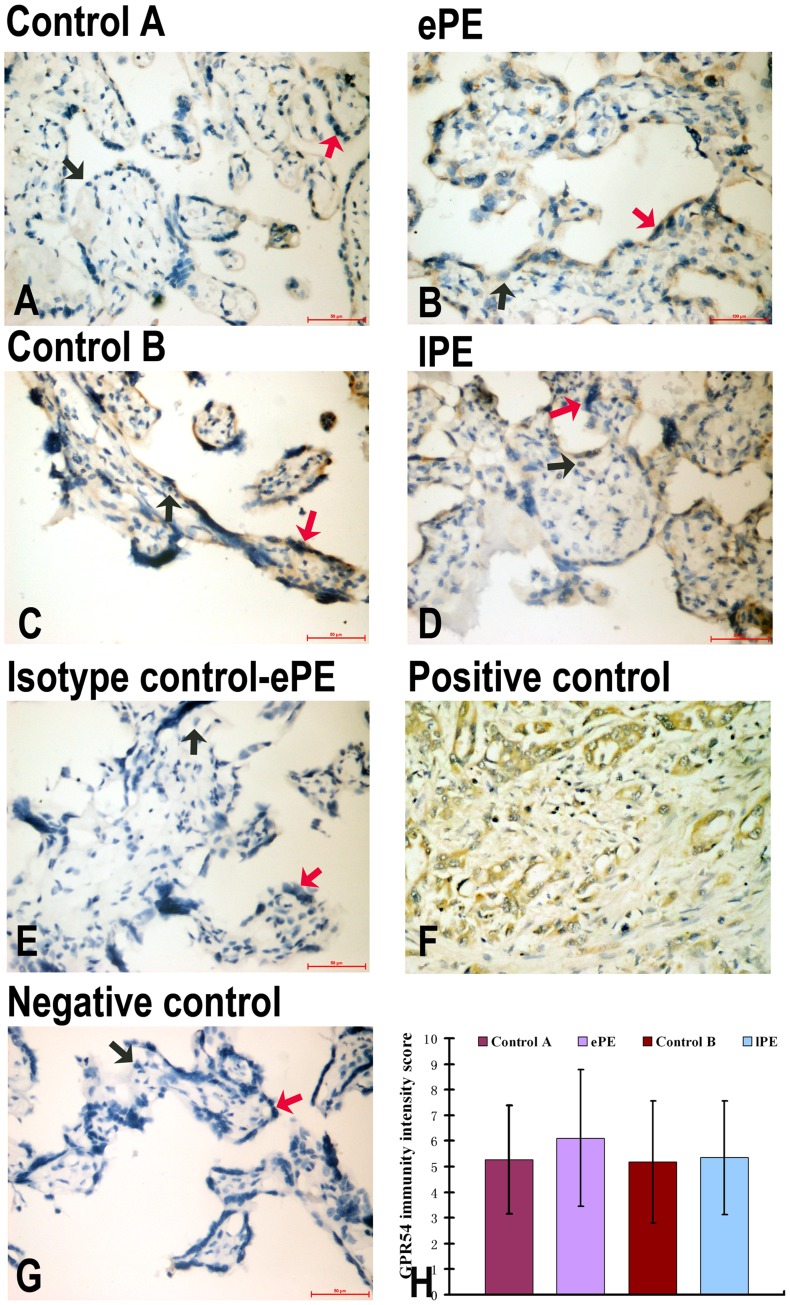
IHC of GPR54 expression in the placentas of Control A (A), ePE (B), Control B (C), lPE (D), isotype control (E), positive control (F), negative control (G). Panel H shows GPR54 immunity intensity score semiquantitation. GPR54 was detected in syncytiotrophoblast from ePE and Control A, and hardly detected from lPE and Control B. Trophoblast from the ePE demonstrated no differences in GPR54 expression with Control A (H). Red arrows indicate the syncytiotrophoblast; black arrows indicate the cytotrophoblast.

### Real-time PCR

Total RNA from 50 mg placental tissue was extracted using Trizol reagent (Invitrogen Life Technologies, China) according to the manufacturer’s instructions. 1% agarose gel electrophoresis was carried out for 30 min to detect the degradation of RNA samples. The concentration and purity of extracted RNA were quantities in a GenQuant RNA/DNA calculator (Amersham-Pharmacia Biotech, Cambridge, UK) Deoxyribonuclease (DNase) treatment was carried out using DNase I (Invitrogen Life Technologies; Carlsbad, CA, USA). Two micrograms of total placental RNA samples was converted to cDNA using the Superscript Reverse transcriptase Kit (Invitrogen Life Technologies,China). The reverse transcription and the following PCR reaction were performed according to instruction. Real-time PCR analysis was then performed to examine the mRNA level of KiSS-1 and GPR54 in placental tissue using an ABI PRISM®7700 sequence detector (Applied Biosystems, Foster City, CA). The qRT-PCRs were carried out using the reagents and protocol contained in the SYBR_GREEN PCR Master Mix (Applied Biosystems, Foster City, CA) and using the following primers: KISS-1(NM_002256) (F) ACT CAC TGG TTT CTT GGC AGC T, (R) CAG AGGCCA CCT TTT CTA ATG G; GPR54 (NM_032551) (F) CGA CTT CAT GTG CAA GTT CGT C, (R) CAC ACT CAT GGC GGT CAG AG. For a quantitative measurement, β-actin (NM_001101) was used as an internal control: (F) ACC AAC TGG GAC GAC ATG GAG AAA A,(R) TAC GGC CAG AGG CGT ACA GGG ATA G. The PCR reaction were carried out in triplicate in a final volume of 25 µl with 2.5 µl (100 ng) cDNA, SYBR® Green Realtime PCR Master Mix 12.5 µl, 0.5 µl sense and antisense primer mixture (10 µM). The specificity of the amplification products was confirmed by melting curve analysis and by agarose gel electrophoresis. The PCR fragments were visualized on 2% ethidium bromide-stained agarose gels. The PCR was performed for 60 seconds at 95°C followed by 40 cycles of 15 seconds denaturation at 95°C and 60 seconds annealing 60°C. The quantity of cDNA for each experimental gene was normalized to the quantity of β-actin cDNA in each sample. Relative expression was determined by using the ΔΔCt (threshold cycle) method according to the manufacturer’s protocol (User Bulletin #2).

**Figure 5:**
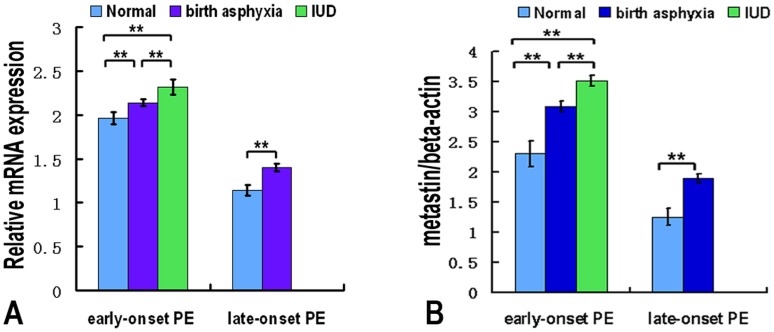
KiSS-1mRNA and metastin expression in placenta samples from different newborn groups of pre-eclampsia. (A). Quantitative RT-PCR analysis of KiSS-1 mRNA expressions in placenta samples from normal newborn, birth asphyxia and intrauterine fetal death (IUD) in preeclamptic pregnancy. (B). Densitometric analysis of metastin protein expressions of preeclamptic pregnancy placenta samples after normalization to β-actin. The results demonstrated not only the transcript level of the KiSS-1 but also the protein level of metastin were significantly higher in the placental sample of IUD and birth asphyxia compared with the normal newborns in ePE and lPE. **P<0.01, significantly increased compared with the respective control.

### Western Blot Analysis

Protein extracts were prepared from placental tissues by homogenization with modified RIPA lysis buffer (50 mM Tris-HCl, 150 mM NaCl, 1% NP-40, 0.25% Na-deoxycholate, 1 mM EDTA, 0.1% SDS) supplement with protease inhibitor cocktail in tablets (Roche diagnostics), and proteins were isolated in an extraction buffer (125 mmmol/L Tris-HCI pH 8.0, 2 mmol/L CaCI_2,_ 1.4% Triton X-100 (v/v) and protease inhibitor cocktail in tablets). Protein concentrations were quantified with the bicinchoninic acid (BCA) protein assay (Thermo Fisher Scientific Inc, Rochester, New York). Protein samples (50 µg) and metastin standard protein (30 µg, Phoenix Pharmaceuticals, Inc. USA) were migrated on a 15% sodium dodecyl sulfate polyacrylamide gel electrophoresis (SDS-PAGE) and transferred on a polyvinylidene fluorid (PVDF) membrane (Amersham Biosciences, Piscataway, NJ, USA). Membrane was blocked with 5% non-fat dried milk in Tris-buffered saline (TBS) with 0.15% Tween-20 and incubated with the primary antibody. The following primary antibodies were used: rabbit anti-human metastin (1–54) Amide (1∶800;Cat. G-048–59, Phoenix Pharmaceuticals, Inc., USA), goat anti-human GPR54 (N-14) (1∶500; Cat. Sc-48219, Santa Cruz Biothenologies, USA), and rabbit polyclonal to β-actin antibody (1∶1000; Cat. Ab8227, Abcam, HK). Primary antibody binding was detected using the following secondary antibody: anti-rabbit IgG and anti-goat IgG antibody conjugated to horseradish perioxidase (1∶10000; Santa Cruz Biotechnologies, USA). Detection was achieved with the ECL chemiluminescence kit (Amersham Bioscience) according to the protocol using X-ray films (Kodak, China).

### Immunohistochemistry

Sections were cut at 5 µm and mounted onto poly-L-lysine coated glass slides. Sections were dried in an oven overnight at 37°C and dewaxed in xylene, rehydrated by passing through descending dilutions of ethanol (100–70%) and rinsed in phosphate-buffered saline (PBS). Sections were treated with 3% hydrogen peroxide in methanol for 10 minutes to block endogenous peroxidase activity before being washed in distilled water for 3× three–minute cycles. Sections were immersed into 0.5% Triton for 30 min followed by three rinses in PBS. Slides were treated with normal goat serum diluted in PBS to prevent nonspecific binding before being incubation in a moist chamber for 60 min at room temperature. Immunohistochemistry was performed using the avidin–biotin complex immunoperoxidase method (Vectostain® Elite ABC Kit; Vector Laboratories, Burlingame, CA). The sections were incubated in a moist chamber for 1 hour at room temperature with KiSS-1 primary antibody (rabbit anti-human metastin (1–54) Amide (1∶400; Cat. G-048–59, Phoenix Pharmaceuticals, Inc., USA) and goat anti-human GPR54 (N-14) (1∶500;Cat.Sc-48219, Santa Cruz Biothenologies, USA). Slides were washed in PBS for 3× three–minute cycles and then incubated further with biotinylated anti-rabbit secondary antibody in PBS (1∶200, Vector Laboratories, Burlingame, CA) for 45 minutes at room temperature. Following another 3× three–minute washes in PBS, slides were incubated with an avidin-biotin-peroxidase complex using the Vectastain Elite ABC kit (Vector Laboratories, Burlingame, CA). After a 45-min incubation at room temperature, the sections were rinsed three times in PBS. For color visualization of the primary antigen–antibody complex, peroxidase substrate solution, (DAB stain kit, Vector Labs, Burlingame, CA) was applied to all sections followed by incubation in a dark, moist chamber for 5 min. After rinsing in tap water, a light hematoxylin counterstain was applied and the sections were dehydrated through graded concentrations of ethanol and cleared through three changes of xylene. Mounting media and coverslips were applied and the sections were examined by light microscopy.

To evaluate metastin and GPR54 staining, we used a case of pancreas tissue as a positive control. This case had been confirmed to show high expression of KiSS-1 and GPR54 by RT-PCR and in situ hybridization (data not shown). We also used rabbit pre-immune (1∶400, Cat. 086199, Life Technologies, USA) and normal goat serum (1∶500, Cat. NS02L-1ML, Merck Millipore, Germany) as primary antibodies for isotype controls. Sections from each sample of human placenta tissue were used as negative controls with the primary antibody replaced with Tris-buffered saline.

### Immunohistochemistry Semi-quantitation

Semi-quantitative analysis of immunohistochemistry of metastin and GPR-54 expression was performed according to a published method [21]. Briefly,10 fields were selected randomly and expression in 1000 cells (100 cells/field) were evaluated using high-power (200×) microscopy. Three investigators (Wang CH, Zhao J and Qiao C) separately evaluated the staining in a blinded manner and the mean values of each investigator’s scores were used for analysis. Specifically, no positive cell was scored as 0, 1–10% of positive cells as 1, 11–50% as 2, 51–80% as 3, and 81–100% as 4. Staining intensity was rated on a scale of 0–3∶0 = negative, 1 = weak, 2 = moderate, and 3 = strong. The percentage of positive cell score was multiplied by the intensity score to obtain a final number for further statistical analysis.

### Statistical Analysis

The gene expression data of quantitative RT-PCR experiments and the western blot analysis, Immunohistochemistry semi-quantitation as well as the clinical data of pregnant women were analyzed for statistical significance by the student’s T test with the program SPSS 16.0 for Windows (SPSS Inc, Chicago IL, USA). Differences with a *P* value <0.05 were regarded as statistically significant.

## Results

### Characteristics of Participants

There were no significant differences in maternal age and delivery age between early and late-onset preeclampsia groups and the matched control group (p≥0.05). There was a marked elevation in systolic and diastolic blood pressure in the preeclampsia groups as compared to the control group (p<0.05).

### Expression of KiSS-1 and GPR54 in Preeclamptic Placenta

We examined 76 pathological placenta for the expression of both genes using quantitative RT-PCR: 36 placenta from women with early-onset preeclampsia (23–33 weeks, ePE) and 40 placenta from women with late-onset preeclampsia (34–39 weeks, lPE). The expression data of the pathological placenta were compared with those of control placenta from matched gestational stages of women with normotensive pregnancies: Control A (23–33 weeks, n = 40) for ePE, and Control B (34–39 weeks, n = 40) for lPE.

The transcript level of KiSS-1 was significantly (p = 0.002) higher in ePE compared to other groups examined. Specifically, the KiSS-1 mRNA level in ePE was upregulated by 2.2- and 1.8-fold as compared to that in Control A and lPE (Fig. 1A and Fig. 1C), respectively. There was no significant KiSS-1 mRNA upregulation in lPE as compared to Control B (Fig. 1A). A significant increase in the level of the KiSS-1 gene product metastin was also seen in the ePE placental tissues compared to the other three groups (Control A, Control B, and lPE) as determined by Western Blot analysis (Figure 2A and B).

In contrast to KiSS-1, there was no significant difference in the level of GPR54 mRNA between ePE and Control A, ePE and lPE), and lPE and Control B (Fig. 1B). No statistically significant difference in the GPR54 protein level was observed among the four groups (Control A, ePE, Control B, lPE (Fig. 2A and C), although there is a trend of higher GPR54 protein levels in both Control A and ePE as compared to Control B and lPE (Fig. 2).

### Localization of Metastin and GPR54 Proteins in Early-onset and Late-onset Preeclamptic Placenta

Expression of metastin was detected in syncytio- and cytotrophoblasts of study groups and the matched controls using immunohistochemistry (Fig. 3 A–D). In Control A and ePE, metastin was most abundantly expressed in syncytiotrophoblasts and moderately in cytotrophoblasts (Fig. 3 A, B). Metastin expression in trophoblasts was significantly increased in ePE as compared to Control A (Fig. 3H), consistent with Western blot results (Fig. 2B). In contrast, metastin expression in syncytiotrophoblasts was not different between lPE and Control B (Fig. 3H), consistent with Western blot results (Fig. 2B).

GPR54 was detected mostly in syncytiotrophoblasts in Control A, ePE, Control B and lPE, but was barely detected in cytotrophoblasts (Fig. 4 A–D), consistent with our Western blot results in Figure 2B. Expression of GPR54 in the trophoblasts of ePE showed no difference as compared to that of Control A, and no difference expression of GPR54 was found between lPE and control B (Fig. 4H).

### The Relationship of Expression of KiSS-1 in Placenta with Birth Asphyxia and Intrauterine Fetal Death

The results demonstrated not only the transcript level of the KiSS-1 (Fig. 5A) but also the protein level of metastin (Fig. 5B) were significantly higher in the placental sample of IUD and birth asphyxia compared with the normal newborns in ePE and lPE, especially in the IUD. The Pearson correlation coefficient of KiSS-1mRNA level and newborn station was 0.467 (p = 0.015) and the Pearson correlation coefficient of metastin level and newborn station was 0.583 (p = 0.026).

## Discussion

This is the first report on the differential expression of KiSS-1 in placenta of ePE and lPE. We demonstrate a significant increase in KiSS-1 expression both at the RNA and protein levels in the placenta of ePE but not of lPE, as compared to gestational-matched controls. We also show that elevated KiSS-1 expression correlates with increased severity of newborn asphyxia.

Abnormal placental development is believed to be more strongly associated with ePE (an onset before 34 weeks of gestation), whereas lPE (an onset at 34 weeks or later) is believed to occur secondary to maternal microvascular diseases, such as long-term hypertension, or to reflect a maternal genetic disposition [9]. Previously we found an increase in the expression of KiSS-1 at both RNA and protein levels in placenta of preeclamptic pregnancies as compared to those of normal term pregnancies [18]. Consistent with our findings, Vasquez-Alaniz et al. investigated the differences in the placental tissue expression of KiSS-1 between 27 preeclamptic women and 27 normoevolution pregnant women by qPCR. They found a higher expression of KiSS-1 in the preeclamptic than the normal control group [22]. An increase of Kisspeptins in serum of patients with preeclampsia was also reported [23]. However, all the studies reported so far were performed using samples from either late-onset preeclampsia or mixed preeclampsia. Thus, it was not clear whether the elevated KiSS-1 expression was derived from ePE, lPE, or both. In addition, the control groups used were normal term pregnancies (gestational weeks of 38–40). In the present studies, we separated preeclampsia into ePE (<34 weeks of gestation) and lPE (>34 weeks) groups and also used gestational age-matched normal pregnancies as the corresponding control groups.

Recently, Cartwright & Williams [24] evaluated mRNA and protein levels of KiSS-1 and KiSS1R in 10 preeclamptic and 10 normal term pregnancy placental tissues by quantitative real-time PCR, western blot and immunochemistry. They found reduced expression of KiSS-1 and increased expression of KiSS1R in preeclampsia compared with control term pregnancy at both the protein and mRNA levels. They also examined first trimester placentas with high versus normal uterine artery resistance using Doppler scanning and found reduced KiSS-1 level in the high resistance group. The apparent discrepancy between these studies and ours is likely due to the following reasons. First, in the Cartwright & Williams studies the case number was very small (10 or 6 in each group). Second, using Doppler to predict preeclampsia is known to have a high false-negative rate [25]. Thus, the possibility that some subjects in the normal resistance group may later become preeclamptic as pregnancy continues could not be excluded.

In the present studies we show that KiSS-1 expression was significantly increased at both RNA and protein levels in ePE, compared to either lPE or gestational age-matched controls. Importantly, there is no significant difference in KiSS-1 expression levels between lPE and the gestational age-matched controls. As KiSS-1 inhibits the migration and invasion of trophoblasts [19], [26] and ePE is associated with trophoblasts shallow invasion and abnormal placental morphology [9], we postulate that up-regulation of KiSS-1 expression may contribute critically to the underlay pathogenesis of ePE and not that of lPE.

Evidence exists that metastin/Kisspeptin (product of KiSS-1) plays an important role in regulating trophoblast invasion through interaction with its receptor GPR54 during the first trimester of pregnancy both in humans and in rodents. We previously reported that KiSS-1 expression in human placental tissues increases gradually with increasing gestational weeks during the first trimester, as determined by a positive linear correlation between KiSS-1 mRNA levels and the gestational days [27]. Similarly, an increase in the mRNA levels of both KiSS-1 and GPR54 in trophoblasts of first trimester placenta in humans and of mice at e12.5 was found to coincide with the time of peak trophoblast invasion when regulation of this process is of critical importance [19]. Further, in vitro studies showed that Kisspeptin-10 inhibits migration and invasion of HTR8SVneo cells, a cell line derived from first trimester human trophoblasts [19], [26]. Together, these studies support a role of KiSS-1 in regulating trophoblast invasion during first trimester.

The mechanisms by which metastin may regulate trophoblast invasion into the decidua have been investigated. In vitro studies showed that KiSS-1 inhibits the activity of the matrix metalloproteinase-9 (MMP-9) [28]. Normally involved in tissue/receptor remodeling, embryo implantation and placental development, the trophoblast-derived MMPs are developmentally regulated throughout pregnancy [8], [29]. Our previous studies demonstrated a higher level of KiSS-1 expression in association with a lower level of MMP-9 expression in the trophoblasts of women with preeclampsia compared with term pregnancy controls [18]. Thus, inhibition of MMP-9 at expression and/or activity by metastin may constitute one potential mechanism [28], [30]. In addition to regulating MMPs, other mechanisms may be involved in metastin-mediated regulation, including activation of focal adhesion kinase [12], [13], [31], alteration of tissue inhibitors of MMPs (TIMPs), as well as the plasminogen activator/inhibitor system.

In the present studies we did not find significant changes in the expression levels of GPR54 in ePE, lPE, or the normal matched controls. This could either reflect a real situation or limitation of our approach. Both trophoblasts and decidua express GPR54 [14], but our analysis did not distinguish which compartment the GPR54 signal was derived from. Thus, possible changes in GPR54 expression in a particular compartment might be masked based on the current method we use to measure GPR54 levels. Future studies will involve more careful measurements of GPR54 levels derived from the separate placental compartments.

The possibility that the observed change in the KiSS-1 expression in ePE might be a consequence of the disease cannot be excluded. It is difficult to rule in or rule out this possibility due to ethical constraints imposed by the time of sample collection at the onset of illness. However, we will further explore the mechanism of KiSS-1 in ePE using animal models with KiSS-1 deficiency or placental tissue samples from humans with mutant KiSS-1 genes in the future.

Finally, we observe that there were 11 cases of birth asphyxia (11/36, 30.56%) in ePE, which was higher than those in lPE (4/40, 10%). There were no birth asphyxia occurred in control group. We only found IUD in ePE not in lPE. It is suggested that newborn poor outcome more common in ePE than in lPE, which may be the results of shallow implantation, poor spiral artery remodel and poor placental circulation in ePE. We also found elevated expressions of KiSS-1 in placenta of intrauterine death and birth asphyxia, in comparison to normal newborns of ePE and lPE. There were no significant differences in KiSS-1 expression at the mRNA and protein level in primary cytotrophoblasts cultured under hypoxic or normal oxygen conditions (data not shown). This suggests that up-regulation of KiSS-1 expression in birth asphyxia and IUD is most likely not a result of hypoxia or placenta ischemia, rather, it is a result of insufficient trophoblast invasion.

In summary, we demonstrate here, for the first time, that KiSS-1 expression is upregulated in ePE, relative to lPE and gestational age-matched controls. Our findings suggest that elevated KiSS-1 expression may contribute to the underlying mechanisms of ePE and also support the concept that ePE and lPE are likely two different entities.
