# Correction: Elevated Expression of KiSS-1 in Placenta of Chinese Women with Early-Onset Preeclampsia

**DOI:** 10.1371/annotation/9887a53a-2b49-4172-8bc6-b63c85e06471

**Published:** 2013-12-16

**Authors:** Chong Qiao, Chunhui Wang, Jiao Zhao, Caixia Liu, Tao Shang

There is an error in the Western Blot for Figure 2A. Kisspeptin54 is incorrectly shown as being 54kD. The correct band size for Kisspeptin54 is 15.4kD. 

